# Allergic diseases and asthma: a global public health concern and a call to action

**DOI:** 10.1186/1939-4551-7-12

**Published:** 2014-05-19

**Authors:** Ruby Pawankar

**Affiliations:** 1 Division of Allergy, Department of Pediatrics, Nippon Medical School, 4F Yayoi, Ichi gokan, 1-4-10, Yayoi, Bunkyo-ku, Tokyo 113-0032, Japan

## 

The prevalence of allergic diseases and asthma are increasing worldwide, particularly in low and middle income countries. Moreover, the complexity and severity of allergic diseases, including asthma, continue to increase especially in children and young adults, who are bearing the greatest burden of these trends. In order to address this major global challenge that threatens health and economies alike it is important to have a global action plan that includes partnerships involving different stakeholders from low-, middle-, and high-income countries.

Allergic diseases include life-threatening anaphylaxis, food allergies, certain forms of asthma, rhinitis, conjunctivitis, angioedema, urticaria, eczema, eosinophilic disorders, including eosinophilic esophagitis, and drug and insect allergies. Globally, 300 million people suffer from asthma and about 200 to 250 million people suffer from food allergies [1]. One tenth of the population suffers from drug allergies and 400 million from rhinitis [1]. Moreover, allergic diseases commonly occur together in the same individual, one disease with the other. This requires an integrated approach to diagnosis and treatment and greater awareness of the underlying causes amongst family physicians, patients as well as specialists

A report from the World Allergy Organization, the *WAO White Book on Allergy (originally published in 2011) and updated in 2013*[1] summarizes the burden of allergic diseases worldwide, the risk factors, impact on quality of life of patients, morbidity, mortality, their socio-economic consequences, recommended treatment strategies, future therapies, and the cost-benefit analyses of care services. It also offers “high level” recommendations for action on allergy education for health care professions and enhanced patient service provision. WAO is concerned about the rising global burden of allergic diseases and is committed to increased cooperation at a global level engaging governments and policy makers to channel resources and efforts to recognizing allergic diseases as a public health issue. WAO has updated the original WAO Book on Allergy, in 2013 to contain new information, providing the latest data, evidence, and treatments with a new chapter on Severe Asthma, updated introduction and executive Summary and several updated chapters.

For instance, asthma prevalence is rising in several high as well as low and middle income countries and the prevalence and impact of allergic diseases continue to grow. According to the World Health Organization, the number of patients having asthma is 300 million and with the rising trends it is expected to increase to 400 million, by 2025. Patients with asthma and allergic diseases have a reduced quality of life. According to the World Health Organization asthma causes 250,000 deaths annually. Moreover, asthma in infancy often goes unrecognized and thus untreated. In the United States, 23 million people including 7 million children suffer from asthma and the prevalence is increasing. The economic costs of asthma are high both in terms of direct and indirect costs [1] (Table 1) especially in severe or uncontrolled asthma. In the United States, pediatric asthma results in 14 million missed days of school each year, which in turn result in lost workdays — and lost wages — for caregivers [2]. As asthma continues to affect more children in lower-income countries, this will lead long-term consequences for their education and perpetuation of their poverty. We need to find ways to control indoor and outdoor air pollution, to train health care professionals to diagnose and treat asthma in children, and to ensure that asthma medications are affordable for all who need them. Educational programs for self-management of asthma and national efforts to tackle asthma as a public health problem have produced remarkable benefits resulting in dramatic reductions in deaths and hospital admissions [1,3].

**Table 1:** The economic burden of allergy

**Country**	**Year costs calculated**	**Population (2010)**	**Disease**	Direct costs*	Indirect costs**	**Total costs estimated**
**Australia**	2007	23 million	All allergies	A$ 1.1 billion	A$ 8.3 billion	A$ 9.4 billion
**Finland**	2005	5.3 million	All allergies	€468 million	€51.7 million	€519.7 million
**South Korea**	2005	50 million	Asthma	-	-	US$1.78 billion
			Allergic Rhinitis			US$266 million
**Israel**		7.5 million	Asthma	-	-	US$250 million
**Mexico**	2007	103 million	Asthma			US$35 million
**USA**	2007	310.2 million	Asthma	US$14.7 billion	US$5 billion	US$19.7 billion
	2005		Allergic Rhinitis	US$11.2 billion	Up tp US$ 9.7 billion	Up to $20.9 billion

A few global facts and figures for two common allergic diseases: asthma and rhinitis.

*Direct costs: Expenditure on medications and health care provision.

**Indirect costs: Cost to society from loss of work, social support, loss of taxation income, home modifications, lower productivity at work, etc.

Extracted from Ref [1]. Pawankar R et al.

The upsurge in the prevalence of allergies is observed as societies become more affluent and urbanized. An increase in environmental risk factors like outdoor and indoor pollution like tobacco smoke combined with reduced biodiversity also contributes to this rise in prevalence. In many low- and middle-income countries including in rural areas in India, people rely on solid fuel (wood, cow dung or crop residues) that they burn in simple stoves or open fires for domestic energy [1]. Secondhand smoke has become more common as parents become affluent enough to buy cigarettes. Together, these factors generate indoor air pollution that is estimated to be as much as 5 times as severe in poor countries as in rich ones [4]. In rural Bangladesh, the prevalence of wheezing in rural children over a 12 month period was 16% [5]. The *White Book* highlights data from China that reports outdoor pollution as a cause of 300,000 deaths annually [1]. Moreover climate change, reduced biodiversity [6], change in ambient temperatures, changes in weather during pollen seasons can cause both biological and chemical changes to pollens and have direct adverse consequences on human health by inducing disease exacerbations especially in urban and polluted regions. Appropriate environmental control measures of risk factors like indoor tobacco smoke, outdoor pollution and biomass fuel can have huge health benefits. There is also other complex but measurable associations between early life circumstances like maternal and childhood nutrition. Such evidences indicate early life opportunities for interventions targeted towards the prevention of allergies and asthma.

Persons with allergic diseases like asthma also often have other comorbid conditions like diabetes, obesity, cardio-vascular disease, gastro esophageal diseases leading to more complex situations and worse outcomes associated with these complications. Furthermore, owing to the high health care costs, morbidity, impact on quality of like, absenteeism, poorer work performance and socio-economic costs, allergic diseases result in a socio-economic burden to the affected families as well as countries. The costs for treating rhinitis in the US have doubled in 5 years to 11 billion US$. In the developed countries, the financial burden of asthma ranges from US$ 300 to 1300 per patient per year annually. In developing countries, like Vietnam it is estimated to be US$184 per patient per year and in India, the monthly cost of medication for an asthmatic child can amount to one third of an average family’s monthly income. In the light of this ever-increasing threat of allergic diseases, high-, middle-, and low-income countries need to come together to develop a common strategy to find solutions at the levels of policy, health care delivery, health communication, and education under a platform of global cooperation. In fact, many developing countries are now caught in a stage of transition in which they face a growing burden of allergic diseases amongst other non-communicable diseases on top of the ongoing health problems of communicable diseases.

Efforts targeting allergic diseases are still very fragmented. The WAO *White Book on Allergy* not only presents data on the growing epidemic of allergy worldwide, but also puts forward a set of recommendations the ***“Declaration of Recommendations*”** targeted towards governments and health care policy makers, 1) need for epidemiological studies to assess the true burden of allergic diseases globally; 2) need to implement appropriate environmental control measures to reduce triggers and risk factors like smoking and outdoor pollutants and develop adequate preventative measures; 3) need to increase the availability of adequate trained personnel to diagnose and treat allergic diseases as well as make provisions for better availability and affordability of drugs; 4) need to bridge the knowledge gap in allergic diseases and asthma leading to increased capacity building; 5) need to increase the clinical expertise in treating allergic diseases and asthma; 6) need to make efforts to increase public awareness and work towards developing innovative preventative strategies.

Global partnerships may encourage rapid and cost-effective scientific innovations. Large multicounty consortia are also needed to provide data from multiethnic populations for studies of genes and epigenetic phenomena, which could unravel the pathophysiological mechanisms behind some noncommunicable diseases; such consortia could also help to develop interventions that promote health globally. While the World Allergy Organization has been making constructive steps in various ways in the last years towards addressing this public health issue, a collaborative effort by the American Academy of Allergy Asthma and Immunology (AAAAI), the European Academy of Allergy and Clinical Immunology (EAACI), the American College of Allergy Asthma and Immunology (ACAAI) and the World Allergy Organization (WAO) called International Collaboration in Asthma, Allergy and Immunology (iCAALL) has been working towards addressing allergic diseases through dissemination of knowledge and raising awareness at various levels.

Globalization is creating an interdependence that affects both the risks of disease and their potential solutions. Global connections are much more apparent in the case of communicable infectious diseases, since viruses and bacteria are more readily perceived as cross-border threats; consequently, these diseases prompt global cooperation, as evidenced by the Global Fund to Fight AIDS, Tuberculosis, and Malaria, among other initiatives. Although we must continue to address these threats, we must also increase the sense of urgency regarding noncommunicable diseases that are “communicated” by means of the global promotion of products and lifestyles, lest they insidiously undermine the health and wealth of nations. We have a great opportunity: global noncommunicable diseases can unite high-, middle-, and low-income countries in a common purpose, given their common causation, increasingly similar mortality rates and economic burdens worldwide, and generalizable preventive and curative solutions. The first challenge, however, will be to energize policymakers to recognize the need and that opportunity. Therefore efforts should be targeted towards a common goal of reducing the burden of allergic diseases, developing cost-effective innovative preventive strategies and a more integrated, holistic approach to treatment thereby preventing premature and unwanted deaths and improving the quality of life of patients.
